# Perspectives of patients, parents, and health care providers on facilitators of and barriers to the transition from pediatric to adult care in inflammatory bowel disease: a qualitative descriptive study

**DOI:** 10.1093/jcag/gwae002

**Published:** 2024-03-15

**Authors:** Allison Bihari, Eytan Wine, Cynthia H Seow, Karen J Goodman, Karen I Kroeker

**Affiliations:** Division of Gastroenterology, Department of Medicine, University of Alberta, Edmonton, Alberta T6G 2X8, Canada; Division of Pediatric Gastroenterology & Nutrition, Department of Pediatrics, University of Alberta, Edmonton, Alberta T6G 1C9, Canada; Division of Gastroenterology, Departments of Medicine and Community Health Sciences, University of Calgary, Calgary, Alberta T2N 4Z6, Canada; Division of Gastroenterology, Department of Medicine, University of Alberta, Edmonton, Alberta T6G 2X8, Canada; Division of Gastroenterology, Department of Medicine, University of Alberta, Edmonton, Alberta T6G 2X8, Canada

**Keywords:** transition of care, pediatric chronic disease, inflammatory bowel disease

## Abstract

**Background:**

The typical transition from pediatric to adult care in patients with inflammatory bowel disease occurs with an increase in health care utilization and a decrease in adherence to medications and scheduled appointments. An effective transition could reduce negative impacts but requires identifying opportunities to improve this process. This study aims to describe barriers and facilitators of transition according to patients, parents, and health care providers.

**Methods:**

This study used a qualitative description approach. The lead author conducted semi-structured interviews with 17 patients, 13 parents, and 15 providers recruited from Western Canada. Latent content analysis identified themes in interview transcripts.

**Results:**

The theme of preparedness emerged across all groups as a transition facilitator. Other facilitators that emerged included patient characteristics, supportive parents, home environment, and supportive adult care team. Themes of barriers that emerged included patient factors, “hovering parents” and family factors, navigating a new health care system, and travel distance.

**Conclusions:**

This study describes facilitators and barriers according to each stakeholder involved in the transition process. Future studies should focus on designing and evaluating interventions aimed at promoting facilitators and addressing identified barriers in patients preparing to transition from pediatric to adult care.

## Introduction

Transition, defined as “the purposeful, planned movement” from pediatric to adult care, is a critical step in the journey of patients diagnosed with a chronic disease in childhood.^1^ While transition is a gradual process of changing care, transfer refers to the actual movement from pediatric to adult care.^2^ During transition, young adults with chronic diseases need to assume responsibility for disease management—a role typically held by their parents. Parents of young adults with chronic diseases simultaneously undergo a transition that requires them to support and facilitate the transfer of responsibility to their child.^3,4^ This is a vulnerable time when patients may experience emotional and psychological challenges as they adapt to a new healthcare team and care setting.^5^ Specifically, in inflammatory bowel disease (IBD) transition typically occurs with an increase in health care utilization [emergency department visits] and a decrease in adherence to scheduled appointments and medication.^6–10^

Canada has one of the highest incidences of childhood-onset IBD in the world.^11^ Further, the number of childhood IBD cases is predicted to triple between 2008 and 2030.^12^ Due to this projected increase in the prevalence of IBD, there is an emphasis on young adults with IBD undergoing appropriate transitioning from the pediatric to the adult healthcare system.^13,14^ Therefore, improving the transition process is currently of great relevance to the field of IBD.

Studies of facilitators of and barriers to transition from pediatric to adult IBD care have typically explored one point of view: that of patients or health care providers. Because transition is a collaborative process involving patients, parents, and providers, comprehensive identification of transition facilitators and barriers requires considering perspectives of all stakeholders.

This study addresses existing limitations by describing reported transition facilitators and barriers according to patients, parents, and providers. This knowledge will be useful for clinicians providing care for transitioning patients and designing transition interventions that acknowledge and address facilitators and barriers from the perspectives of diverse stakeholders.

## Methods

### Study design and aims

The current analysis is part of a study for which the primary author (A.B) conducted interviews with the primary aim of defining IBD transition success from the perspective of patients, parents, and providers.^15^ This analysis used a qualitative descriptive approach to provide a rich account of the phenomenon of transition from pediatric to adult care.^16,17^ This approach allowed the authors to address the additional research question: “What are the facilitators of and barriers to achieving a successful transition according to patients, parents, and health care providers?”. The study was approved by all site research ethics boards (University of Alberta: Pro00099184; University of Calgary: REB20-0979; University of British Columbia: H20-01722). We used the standards for reporting qualitative research to guide the reporting of results.^18^

### Settings and recruitment

We recruited IBD providers, patients, and their parents from IBD clinics in Edmonton and Calgary, Alberta, Canada using purposive and snowball sampling. Providers were additionally recruited from British Columbia. Details of participant recruitment are published.^15^ We included patients if they transferred to adult care within the preceding two years and had a known diagnosed of IBD for at least a year before transfer. Providers were included if they had at least one year of experience caring for IBD patients. Patients were excluded if they had comorbidities unrelated to IBD.

### Trustworthiness

Efforts to ensure trustworthiness were guided by criteria from Lincoln and Guba^19^ and are outlined in the Supplementary Material.

### Data collection and analysis

Interview guides were developed for each stakeholder group with consultation from an expert in qualitative research. IBD clinicians (E.W, C.S, K.K) reviewed the guides for content. A.B conducted interviews virtually between June 2020 and March 2021. Interviews were recorded and transcribed verbatim. A.B used latent content analysis to analyze interviews with NVivo 1.2.^20^ Latent content analysis uses a process of identifying, coding, and categorizing patterns within discursive data from transcribed interviews until themes emerge.^21^ A.B analyzed interviews concurrently with data collection and coded interviews independently within each stakeholder group. Interviews were analyzed simultaneously for the primary aim of defining transition success and for the aims specific to this study. For this study, questions analyzed included asking each stakeholder to describe facilitators of and barriers to either their/their child’s/patient’s transition (when applicable) and then for transition in general. Participant recruitment concluded when saturation was achieved for the study’s primary aim.

## Results

### Participants

**Figure 1.**
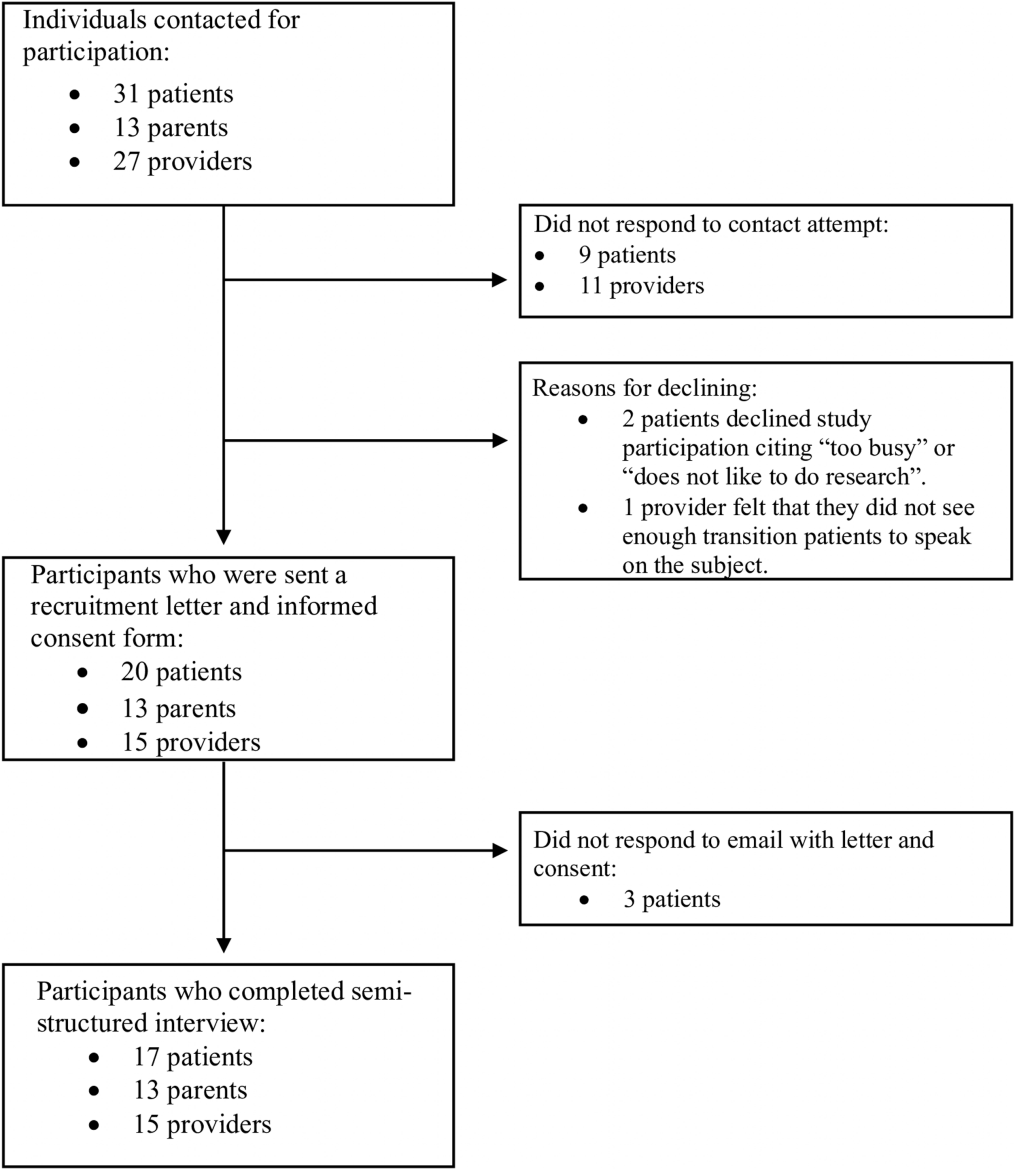
Flowchart of participant recruitment.

A total of 31 patients, 13 parents, and 27 providers were initially contacted for study participation. Of these stakeholders, 9 patients and 11 providers could not be reached, while 5 patients and 1 provider declined study participation. In total 17 patients, 13 parents, and 15 providers participated in an interview. Figure 1 provides a flowchart of participant recruitment. Participant demographics have been outlined.^15^ In brief, 58.8% of patients were female sex, 52.9% had Crohn’s disease, and 58.8% were diagnosed between the ages of 15 and 17. Further, 29.4% of patients were on biologic medications; 17.6% were on either 5-ASA or an immunosuppressant; 41.1% were on a combination biologic; 5.9% were not on any medication. Although not directly asked, three patients mentioned having had surgery for their IBD. All 13 parents were mothers of IBD patients. 10 parent-patient dyads participated, and 61.5% of parents did not have their IBD child living with them at the time of the interview. Providers included five adult gastroenterologists, two pediatric gastroenterologists, six adult IBD nurses, and one pediatric IBD nurse.

### Facilitators

**Figure 2.**
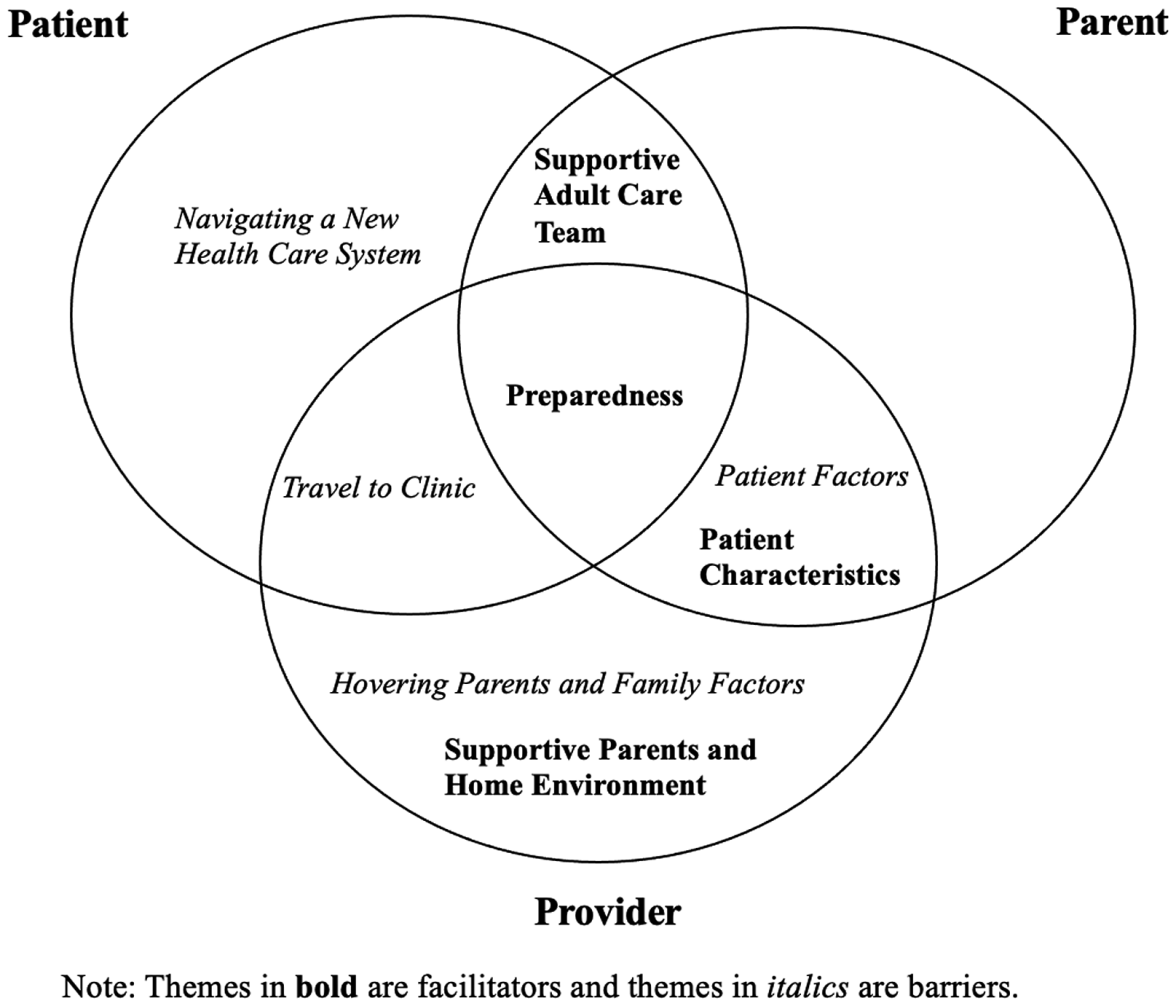
Venn diagram of themes of **facilitators** of and *barriers* to a successful transition according to patients, parents, and providers.

We asked stakeholders to describe facilitators of a successful transition. Themes included preparedness, a supportive adult care team, patient characteristics, and supportive parents and home environment. Facilitator themes are represented in **Figure 2** and quotations are in **Table 1**.

**Table 1.** Quotations of facilitators of and barriers to transition.

Themes	Stakeholder (*n*)	Quotations
*Facilitators*		
Preparedness	Patients (7)	“I think the most important is just asking a lot of questions and if you’re uncomfortable with the process making sure that there is someone that you can ask questions to and that someone can answer those questions…”
	Parents (7)	“I think that [transitioning patients] could use some counselling or something [that] could have helped especially in the transition when he was 18/19 trying to cope with this stuff on his own now.”
		“I think [the patient being] mentally prepared. Some kids are totally mentally prepared and making their own appointments and some kids just aren’t.”
	Providers (6)	“How well they have been set up on the other side. A lot of the kids come over from the pediatric GIs and some of the kids seem better set up by the person who set them over.”
Supportive adult care team	Patients (10)	“I think that the [adult] clinic somehow needs to put a little extra attention onto kids or young adults because you do get busy and forget things and you’re stressed out going to school… so I think like a scheduled 3-month call would be nice for a lot of people to remind them like *‘hey we are here if you’re having issues. How are your symptoms? How have they been this last season?’*” “[The] new doctor being in the same hospital already made it easier.”
	Parents (4)	“…even just someone at the clinic - maybe the GI clinic has a liaison person and that person’s job is to check up on these kids that are coming out of [pediatric care].” “… give [the transitioning patient] a year to adjust because they are used to an entirely different approach.”
Patient characteristics	Parents (4)	“…a positive attitude and being willing to understand more about the importance of acknowledging symptoms, following up with physician when you have symptoms and respecting the disease I guess is the best way to say it...”
	Providers (9)	“A little bit of less reliance on their parents and taking their own control of their own health, reporting their own symptoms, right?”
		“It depends on their maturity levels as well and whether they’re usually independent at that age and use to undertaking initiative to looking after their own affairs.”
Supportive parents and home environment	Providers (8)	“Parents who are just there to listen and act as a fly on the wall are certainly much easier for the patient overall.”
		“I think if the patient has a stable home environment where they feel confident, where they feel supported, where they have the ability to make some decisions on their own…”
*Barriers*		
Patient factors	Parents (4)	“[Young adults] just aren’t good about advocating for themselves that they are lost in the system a bit or they can potentially get lost.”
	Providers (8)	“I think [transitioning patients] are young and they think they’re invincible and they haven’t really- it’s just a lack of knowledge of their disease and what is involved in maintaining remission and avoiding the progression.” “When there are mental health issues, I think that that can make it quite challenging so anxiety, depression can really make it challenging.”
Hovering parents and family factors	Providers (11)	“A lot of the families that we do see struggle often [because] they have so much other stresses and factors on their plate that it’s hard for them.” “The parents may be overprotective and not interested in supporting youth independence.”
Navigating a new health care system	Patient (11)	“There was a lot of follow up all the time, but then I switched to an infusion clinic and you’re nowhere near a doctor’s office or if you have questions or concerns there is no one to talk to…” “I think a big barrier was it was very intimating. That was the biggest thing. I was just terrified. I didn’t feel super comfortable about the adult setting because it is very strange, I think.”
Travel to clinic	Patient (5)	“…moving cities is really hard and then you’re also really young trying to do things on your own. My parents weren’t involved at all so I was the one transferring different cities and my healthcare and everything like that. You don’t really know how to do that – how to make those phone calls, how to find doctors, how to find referrals is all new.” “I mean the only issues are that I don’t drive so sometimes getting around is a little tough because I also don’t live at home anymore…”
	Provider (4)	“… [transitioning patients] are going into post-secondary education and often they’re moving away to different places and then you have to hand off care to someone else and that again [is] another point they can fall off in terms of follow up from that aspect.”
		“I certainly think if they live further away from a center where their practitioner is – that definitely makes it more difficult.”

#### Preparedness

Patients (7/17), parents (7/13), and providers (6/15) mentioned preparedness for transition as a facilitator. Patients gave examples of preparedness such as patients speaking for themselves in pediatric care, asking questions about adult care, knowing the differences between pediatric and adult care, and being able to report their disease management/history accurately.

Parents suggested that there be an overlap between pediatric and adult care and/or their child having a visit with the adult care team before transferring to adult care. They pointed out that an overlap would allow for transitioning patients to feel prepared for adult care. Parents also described being mentally prepared for changing care. One parent mentioned how their child’s pediatric doctor talked positively about the adult doctor, which helped make them and their child feel more prepared to transition. Three parents suggested that there be a peer support program so patients could ask questions to patients who already transitioned. Parents suggested that counseling be available to help transitioning patients cope with changes in care.

Providers defined preparedness as patients knowing about their disease and how the pediatric and adult care systems differed. One provider mentioned that prepared patients would know the expectations of patients in adult care (eg, attending appointments independently).

#### Supportive adult care team

Ten patients and four parents mentioned the adult care team as a facilitator. Patients gave examples such as feeling that their adult care team cared about them, made them feel welcome, and ensured that they understood their care plan. Patients described a supportive adult team as providing regular follow-up to the patient, maintaining open lines of communication, and being responsive to the patient’s questions. Logistic facilitators included an adult team who offered the patient a first visit before their 18th birthday and an appointment routine that was like their pediatric appointment routine. Patients described the location of their adult care appointments as a facilitator. For example, appointments that were in the same hospital as their pediatric clinic or easy to get to with public transportation were described as facilitators.

Parents described a supportive adult care team as understanding that transitioning patients needed to adjust to the new care system and providing patients with information and resources on who to call and how to arrange health insurance. The adult clinic being in the same location as the patient’s pediatric clinic was described as facilitating better communication between the two systems and being easier for the patient to access.

#### Patient characteristics

Nine providers and four parents described patient characteristics as facilitators. Providers mentioned maturity, having organizational skills, demonstrating independence to make decisions, and prioritizing their health needs independently from their parents as facilitators. Parents described the ability to reach out to the care team, make health decisions, identify symptoms, and having organizational skills and a positive attitude about the transition.

#### Supportive parents and home environment

Eight providers emphasized supportive parents and the home environment as facilitators. Providers described supportive parents and the home environment as a family system that promoted the patient’s self-confidence and encouraged the patient to advocate for themselves, and where the patient had an open relationship with their family to discuss mistakes, could make their own decisions with limited parental inference, and where parents promoted their child’s independence in managing their health.

### Barriers

We asked stakeholders to describe barriers to a successful transition. The themes that emerged included patient factors, hovering parents and family factors, travel to the clinic, and navigating a new health care system. Only 10/13 parents felt that they could provide input on barriers based on their experience. Barrier themes are represented in Figure 2 and quotations are in Table 1.

#### Patient factors

Patient factors emerged in eight provider and four parent interviews. Four providers mentioned mental health comorbidities, such as anxiety and depression, as barriers to transition. Providers described barrier characteristics as being unorganized, irresponsible, in denial of their disease extent, and uninformed about IBD progression, medications, and the importance of disease management, as well as having fatigue related to their disease activity and engaging in substance use. One provider mentioned that a patient with well-controlled disease may not recognize the importance of disease management.

Parents emphasized similar factors, such as being unable to self-advocate, not feeling comfortable asking questions, or reaching out to their care team and lacking maturity.

#### Hovering parents and family factors

Twelve providers mentioned the transitioning patient’s family as a barrier. Providers gave examples of parents who were “hovering and overbearing," reluctant to allow their child to become responsible for disease management, and who speak on their child’s behalf during appointments. Other examples included families who experienced economic and personal challenges and unstable families. One provider mentioned the potential for cultural barriers and emphasized the need to understand family dynamics in different cultures and how these dynamics may impact the family’s ability to transfer responsibility for disease management and promote independence in their child.

#### Navigating a new health care system

Eleven patients mentioned the need to navigate the new adult care system as a barrier. Two patients commented on differences in infusion clinics between pediatric and adult care, with one patient saying “…then I switched to an [adult] infusion clinic and you’re nowhere near a doctor’s office or if you have questions or concerns there is no one to talk to.” Patients mentioned having to figure out transportation to the IBD clinic, organizing referrals, and feeling intimidated by the adult care system.

#### Travel to clinic

The need to travel to the IBD clinic emerged in five patient and four provider interviews. Patients gave examples of moving away from school, living far from the IBD clinic, or not driving and having to rely on local transportation or rides from others to get to their appointments.

## Discussion

To the best of our knowledge, this is the first multi-center study in Canada to describe facilitators of and barriers to patients transitioning from pediatric to IBD adult care according to patients, parents, and health care providers. Engagement of all stakeholders in transition allows for the assessment of similarities and differences in barriers and facilitators identified by the stakeholder groups.

A systematic review conducted by Gray et al (2018) summarized facilitators of and barriers to transition in chronic diseases.^22^ IBD studies in this review showed overlap with our themes of hovering parents and family factors and patient characteristics.^23,24^ A study of transition barriers that interviewed IBD providers in the United States identified themes of helicopter parenting and patients’ developmental maturity.^25^ Nearly all (11/12) providers in this US study gave the example of parents who wanted to maintain control of their child’s care. The characterization of parents wanting to maintain control as a barrier was prominent in our study where 11/15 providers described “hovering parents” as a barrier. The theme of patients’ developmental maturity that arose in the US study was like themes focused on the importance of patient maturity that emerged in our study. The US study found barriers to include patients being inadequately prepared by the pediatric team whereas in our study, patients’ preparedness to transition emerged as a facilitator.

Gray et al (2015) conducted focus groups with patients, parents, and health care professionals about the transition to IBD adult care in the United States.^24^ The theme of high parental involvement, like the theme of hovering parents and family factors in our study, emerged as a barrier in the focus groups. The theme of over-parental involvement emerged in all groups in the focus group study, whereas in our study, this theme only emerged in the provider group. One possible explanation for this difference is that the focus group study asked specifically about “concerns with transitioning,” while our study asked about “barriers.” Parents and patients may be hesitant to acknowledge parental over-involvement as a barrier, but they may be more likely to acknowledge parental involvement as a concern. In the focus group study, the theme of receiving poor quality of care emerged. The theme of poor quality of care overlaps facilitator themes that emerged in our study, including a supportive adult care team and the need to navigate a new system. Other themes from the focus group study that did not emerge in our study included finance and the loss of relationships with the pediatric team that transition entails. The first theme may be more related to the private healthcare system landscape in the United States compared to the publicly funded system in Canada. In the focus group study, the second theme of loss of relationships emerged within the pretransfer group only and not the already transferred group, whereas our study only included patients who already transferred.

Our study reveals similarities and differences between how different stakeholder groups characterize facilitators of and barriers to transition. The only theme to emerge across all groups as a facilitator was preparedness. Concordance on this facilitator supports the need to develop guidelines and resources to ensure patients are prepared to transition.

Our finding that different themes emerged across diverse stakeholders shows the need to engage patients, parents, and providers to achieve a comprehensive characterization of facilitators of and barriers to transition. For example, most providers viewed patients as potential barriers, but this theme was mentioned by four parents and no patients. Patients may be reluctant to recognize themselves as a detriment to their transition and more inclined to focus on the responsibility of the adult care system, as reflected by the themes of navigating a new health care system and having a supportive adult care team. Patients and parents may have been less likely to view mental health comorbidities, mentioned by providers, as a barrier because none of the patients in our study experienced a mental health comorbidity. Although a few patients and providers acknowledged the impact that a patient moving away may have on their transition, this theme did not emerge in the parent interviews. Parents of patients who move out may have been less involved in their child’s care and thus, did not see this as a barrier.

Patients were the only group to mention having to navigate the adult care system as a barrier to transition. During the transition, patients are the primary group taking responsibility and navigating the adult care system, which may explain why this theme only emerged in the patient group. Providers can help patients understand the differences between the pediatric and adult systems with the goal of developing patients’ self-confidence in navigating the new care system.

Providers were the only group to mention aspects of the patient’s family as both a facilitator and barrier, shown by the themes of supportive parents and home environment and hovering parents and family factors. Future research could explore this discrepancy to find out the extent to which patients view their parents’ involvement during their transition as detrimental or helpful.

Study limitations include the potential for selection bias; parents and patients who viewed transition favourably may have been more likely to participate than those who viewed their transition unfavourably. Additionally, this study only represents the viewpoints of mothers; however, the exclusive participation of mothers is consistent with the literature on parental involvement in their child’s chronic disease.^26–28^ As ~70% of patients were either on a biologic or combination biologic, this study likely reflects the viewpoints of patients with moderate–severe IBD. Further, there were no trends of themes emerging within groups based on specific characteristics. For example, 6/7 patients on combination biologic mentioned the theme of a supportive adult care team, but the theme also emerged in 3/3 of patients on 5-ASA or immunosuppressants. The lack of trends between themes and characteristics may be reflective of a low sample of stakeholders with a specific characteristic. Because this study did not collect information on sociocultural data, we cannot assess how representative the study population was of IBD cases in Alberta. The parents and patients interviewed may have been from a higher socioeconomic class and, if so, it could be for this reason that they did not view finances as a barrier to transition. Further, it is not clear if the characteristics of participants prevented cultural differences from emerging as a facilitator or barrier.

The strength of this study is that it describes transition facilitators and barriers according to the stakeholders involved in the process of transition. Future research could focus on designing transition interventions that promote facilitators while addressing the barriers identified in this study.

## Supplementary data

Supplementary data are available at *Journal of the Canadian Association of Gastroenterology* online.

gwae002_suppl_Supplementary_Materials

## Data Availability

The data underlying this article will be shared on reasonable request to the corresponding author. Interview guides used in this study are available in the supplementary material of our article, “defining transition success for young adults with inflammatory bowel disease according to patients, parents and health care providers.”, at https://doi.org/10.1093/jcag/gwac004 [doi].
