# Supplementary material for: Perspectives of patients, parents, and health care providers on facilitators of and barriers to the transition from pediatric to adult care in inflammatory bowel disease: a qualitative descriptive study
Source: J Can Assoc Gastroenterol. 2024 Mar 15;7(3):269–76. doi: 10.1093/jcag/gwae002 (PMC11149662; doi:10.1093/jcag/gwae002)
Supplement: gwae002_suppl_Supplementary_Materials [file gwae002_suppl_supplementary_materials.zip › gwae002_suppl_Supplementary.docx]

**Supplementary Material**

Lincoln and Guba suggest the criteria of credibility, transferability, dependability, and confirmability for promoting trustworthiness in qualitative research.

The following list outlines the steps taken to ensure each of the above criteria was met:

| **Criteria** | **Steps taken** |
| --- | --- |
| Credibility | *Peer debriefing:* Regular discussions with the authors focused on ensuring plausibility of the themes and to limit the influence of the coding author’s biases on the formation of codes and themes.  *Member checking:* During interviews A.B verified interpretations with the participants to ensure accuracy in meaning. |
| Transferability | *Rich description:* A detailed description of participants and settings of the interview was provided in both this manuscript and in the original article. |
| Dependability | *Audit Trail:* Decisions, choices, and insights related to the study were documented, including materials, such as interview transcripts. |
| Confirmability | *Reflexivity:* A.B engaged in field notes directly after each interview and focused on documenting interview setting, overall impression, and any opinions and bias forming from the interview. Additional efforts to practice self-reflexivity included the documentation of A.B’s positionality statement. |

**Positionality Statement**

The first author (A.B) is a female, who was in her mid-20s at the time of conducting the interviews and had taken a course on qualitative research during her graduate degree. A.B approached this research from an outsider perspective, not having experience with a chronic disease, being a parent, nor a health care provider. A.B did however have experience with the transition to becoming a young adult and moving out to pursue post-secondary schooling; therefore, self-reflexivity efforts focused on acknowledging personal opinions on barriers and facilitators to young adulthood to prevent its influence on the data. Further, A.B had no prior relationship with any of the study participants. E.W, C.S, and K.K had prior relationships with the providers interviewed (colleagues) and patients in their role as clinicians. To prevent the potential influence of these relationships on the data, none of these authors were involved in the data collection and participants were made aware of the confidentiality of their answers. Ongoing meetings with the study team ensured that personal opinions on barriers and facilitators as clinicians caring for transitioning patients had limited influence on the data, and served to confirm the plausibility of the themes, but not the creation of themes.
